# Potentially Toxic Elements and Male Reproductive Toxicity: Mechanisms, Implications, and Protective Strategies: A Narrative Review

**DOI:** 10.1002/jbt.71128

**Published:** 2026-10-11

**Authors:** Sakshi Yadav, Placheril J John

**Affiliations:** ^1^ Department of Zoology IIS (deemed to be) University Jaipur Rajasthan India

**Keywords:** male infertility, nutraceuticals, oxidative stress, testicular injury

## Abstract

The male reproductive system is highly vulnerable to environmental toxicants, particularly potentially toxic elements (PTEs), which represent a growing public health concern worldwide. Increasing evidence indicates that these elements impair male reproductive function through interconnected mechanisms including oxidative stress, endocrine disruption, inflammation, apoptosis, and DNA damage, resulting in impaired spermatogenesis and infertility. This review summarizes the current understanding of the major PTEs associated with male reproductive toxicity and highlights the underlying cellular and molecular mechanisms responsible for testicular injury. In addition, it discusses emerging protective and therapeutic strategies, including antioxidants, phytochemicals, nutraceuticals, chelating agents and lifestyle‐based interventions that have shown potential in mitigating PTE‐induced reproductive damage. A comprehensive understanding of these mechanisms and protective approaches may facilitate the development of effective preventive and therapeutic strategies to preserve male reproductive health in populations exposed to PTEs.

## Graphical abstract

Potentially toxic elements impair male reproductive function through oxidative stress, DNA damage, mitochondrial dysfunction, inflammation, endocrine disruption and epigenetic alterations. Antioxidants, phytochemicals, chelating agents and healthy lifestyle interventions may help mitigate testicular toxicity and preserve fertility.

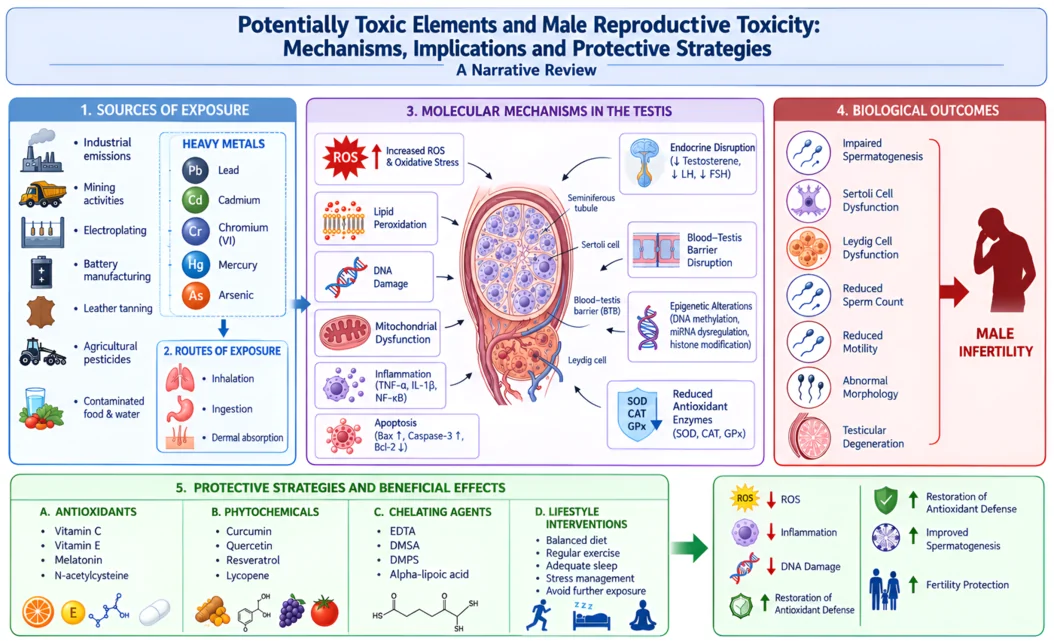

## 1 Introduction

Potentially Toxic Elements (PTEs) are persistent environmental contaminants that pose a major threat to ecosystems and human health. Being non‐biodegradable, they accumulate in biological tissues and in some cases, undergo bio magnification through food chains [1]. Although certain PTEs are essential for normal physiological and biochemical processes at trace concentrations, excessive exposure can result in toxicity and adverse health effects [2]. The term “heavy metals” (HMs) has historically been widely used in environmental toxicology, but because it lacks a universally accepted scientific definition, the term “PTEs” [3, 4] is used preferentially throughout this review, except when referring directly to previously published studies.

Rapid industrialization, urbanization and intensive agricultural practices have substantially increased environmental contamination by PTEs such as lead (Pb), cadmium (Cd), chromium (Cr), mercury (Hg), and arsenic (As) through mining, smelting, electroplating, fossil fuel combustion, pesticide application, and industrial waste disposal [5].

Among the various organ systems affected by PTE exposure, the male reproductive system is particularly vulnerable because of the high content of polyunsaturated fatty acids (PUFA) in sperm membranes, limited cytoplasmic volume and the relatively limited antioxidant defenses of spermatozoa [6]. Consequently, occupational and environmental exposure to these elements has been associated with impaired semen quality, hormonal disturbances and reduced male fertility, contributing to the increasing global burden of reproductive disorders [7]. Given the worldwide decline in male reproductive health, understanding the effects of PTE exposure has become an important public health and environmental concern.

Over the past decade, experimental, clinical and epidemiological studies have considerably advanced our understanding of PTE‐induced male reproductive toxicity. However, important knowledge gaps remain regarding the effects of chronic low‐dose exposure, mixed‐metal toxicity, individual susceptibility, long‐term reproductive outcomes and the translation of findings from experimental models to humans. Furthermore, although several therapeutic and preventive interventions have shown encouraging results in experimental studies, their clinical applicability and long‐term effectiveness require further investigation. This review summarizes the major PTEs, their mechanisms of testicular toxicity, experimental and epidemiological evidence, protective strategies and future research priorities.

## 2 Materials and Methods

This narrative review synthesized relevant literature published between 2011 and 2026. Literature was identified through searches of PubMed, Scopus, Google Scholar and other relevant scientific databases using combinations of keywords related to PTEs, male reproductive toxicity, testicular toxicity, oxidative stress, cadmium, lead, chromium, mercury, and arsenic. Additional relevant studies were identified from the reference lists of retrieved articles. No formal systematic‐review protocol or meta‐analysis was undertaken.

### 2.1 Major Sources of PTE Exposure

Major sources of PTEs in the environment originate from both natural processes and anthropogenic activities (Figure 1). Natural sources include weathering of rocks, volcanic eruptions and soil erosion, which gradually release metals into the air, water, and soil [8]. However, anthropogenic activities contribute substantially more to environmental contamination, particularly mining, industrial manufacturing, fossil fuel combustion, waste disposal, and the extensive use of pesticides and fertilizers. Vehicle emissions and improper disposal of industrial and electronic waste further contribute to environmental contamination [9].

**Figure 1:**
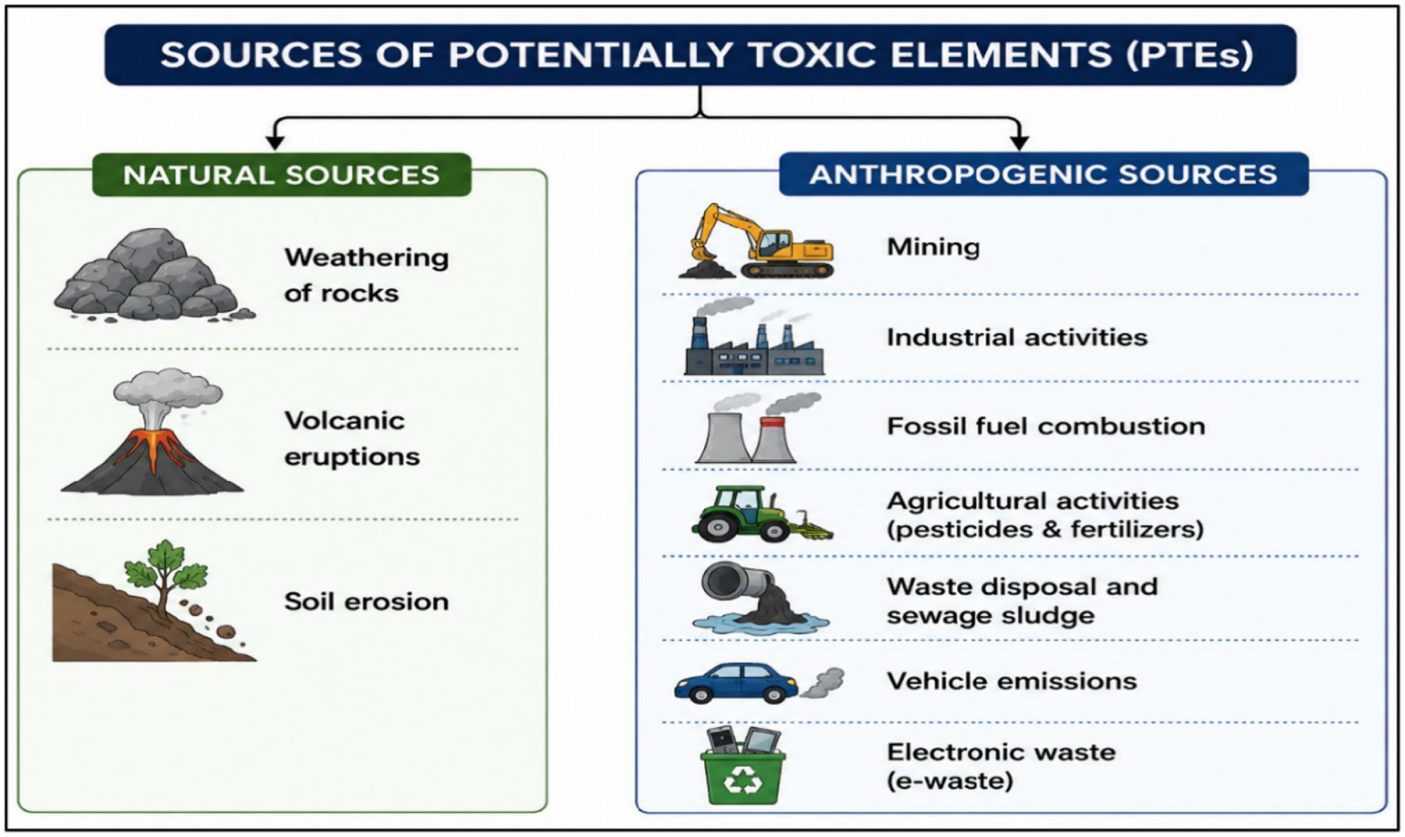
Major sources of human exposure to PTEs.

Human exposure to PTEs has increased markedly since the rapid expansion of industrialization during the 20th century. As a result, they accumulate in living organisms and exert harmful effects on multiple tissues and organs [10]. In addition to industrial emissions, various consumer products, including paints, cosmetics, pesticides and herbicides, may also contain PTEs, contributing to chronic human exposure [11].

Agricultural practices are another important source of PTE exposure. Excessive use of pesticides, sewage sludge and phosphate fertilizers contaminates agricultural soils with metals such as Cd and As, which are subsequently taken up by crops. Consequently, consumption of contaminated cereals, grains and vegetables represents a major dietary exposure pathway, particularly in developing countries [11]. Environmental contamination of air, water and soil further increases exposure risks. Drinking water contaminated with As or industrial effluents containing Hg and Pb has been associated with reproductive toxicity in exposed populations [12].

Lifestyle factors also contribute to PTE exposure. Cigarette smoke is a well‐recognized source of Cd and Pb, while alcohol consumption may enhance metal absorption and exacerbate their toxic effects. Furthermore, the use of traditional medicines, cosmetics and inadequately regulated herbal supplements has also been identified as a potential source of PTE ingestion [13].

Environmental concentrations of PTEs vary considerably depending on geographical location and anthropogenic activities. According to the World Health Organization (WHO) Guidelines for Drinking‐water Quality, the recommended maximum concentrations in drinking water for lead, cadmium, arsenic, inorganic mercury and total chromium in drinking‐water are 10, 3, 10, 6, and 50 µg/L, respectively [14]. Although environmental levels are generally low, chronic exposure through contaminated food, water and occupational settings results in cumulative bioaccumulation and increased reproductive risk.

### 2.2 General Mechanisms of PTE‐Induced Male Reproductive Toxicity

PTEs have harmful impacts on male reproductive health via various, frequently interconnected molecular and cellular processes (Figure 2). They disrupt redox balance, hormone signaling, cellular stability and genetic control, thereby affecting spermatogenesis and fertility [15].

1. **Oxidative Stress and Excessive ROS Production**: PTEs promote reactive oxygen species (ROS) generation through mitochondrial electron transport chain (ETC) dysfunction, Nicotinamide Adenine Dinucleotide Phosphate (NADPH) oxidase activation and depletion of endogenous antioxidants. Excessive ROS damages membrane lipids, proteins and nucleic acids while suppressing antioxidant enzymes including superoxide dismutase (SOD), catalase (CAT) and glutathione peroxidase (GPx). Persistent oxidative stress activates redox‐sensitive signaling pathways, including nuclear factor kappa B (NF‐κB), while perturbing Nuclear Factor Erythroid 2–Related Factor 2 (Nrf2)‐mediated antioxidant responses, thereby contributing to inflammation, oxidative damage and apoptosis [16].
2. **Endocrine Disruption and Steroidogenic Dysfunction**: PTEs impair steroidogenesis by downregulating steroidogenic acute regulatory protein (StAR), cytochrome P450 family 11 subfamily A member 1 (CYP11A1), 3β‐Hydroxysteroid dehydrogenase (3β‐HSD) and 17β‐Hydroxysteroid dehydrogenase (17β‐HSD) expression in Leydig cells, thereby reducing cholesterol transport into mitochondria and testosterone biosynthesis. In addition, disruption of the hypothalamic–pituitary–gonadal (HPG) axis alters luteinizing hormone (LH) and follicle‐stimulating hormone (FSH) secretion, further impairing spermatogenesis [17].
3. **Activation of Apoptotic Pathways in Testicular Cells**: ROS‐mediated mitochondrial damage increases Bax expression while reducing Bcl‐2 levels, leading to mitochondrial outer membrane permeabilization, cytochrome c release, apoptosome formation and activation of caspase‐9 followed by caspase‐3 [18]. In parallel, some PTEs activate death receptor pathways involving Fas/FasL and caspase‐8, accelerating germ cell apoptosis [19].
4. **Ionic Mimicry and Disruption of Cellular Homeostasis**: Certain PTEs, particularly Pb^2+^, can mimic essential ions such as Ca^2+^ and interfere with ion‐dependent cellular signaling [20]. In reproductive tissues, such disturbances may affect processes including steroidogenesis and sperm function.
5. **Damage to the Blood–Testis Barrier (BTB)**: PTEs disrupt Sertoli cell tight junction proteins including occludin, claudin‐11 and zonula occludens‐1 (ZO‐1), increasing BTB permeability and facilitating inflammatory cell infiltration into seminiferous tubules [21, 22].
6. **Genotoxic Effects and Sperm DNA Damage**: ROS induce oxidative DNA damage by forming 8‐hydroxy‐2′‐deoxyguanosine (8‐OHdG), DNA strand breaks and chromosomal instability. Since sperm have limited DNA repair capacity, these lesions impair sperm function and reduce fertility [23].
7. **Inflammatory Responses and Cytokine Imbalance**: PTEs activate NF‐κB signaling through ROS‐dependent phosphorylation of inhibitor of κB alpha (IκBα), leading to increased expression of tumor necrosis factor‐alpha (TNF‐α), interleukin‐1 beta (IL‐1β), interleukin‐6 (IL‐6) and inducible nitric oxide synthase (iNOS). These inflammatory mediators further enhance oxidative stress and impair spermatogenesis [24].
8. **Epigenetic Alterations and Transgenerational Effects**: Emerging evidence suggests that PTEs alter DNA methylation, histone modifications and microRNA expression, thereby dysregulating genes involved in spermatogenesis, steroidogenesis and antioxidant defense. Such epigenetic alterations may contribute to transgenerational reproductive toxicity [25].

**Figure 2:**
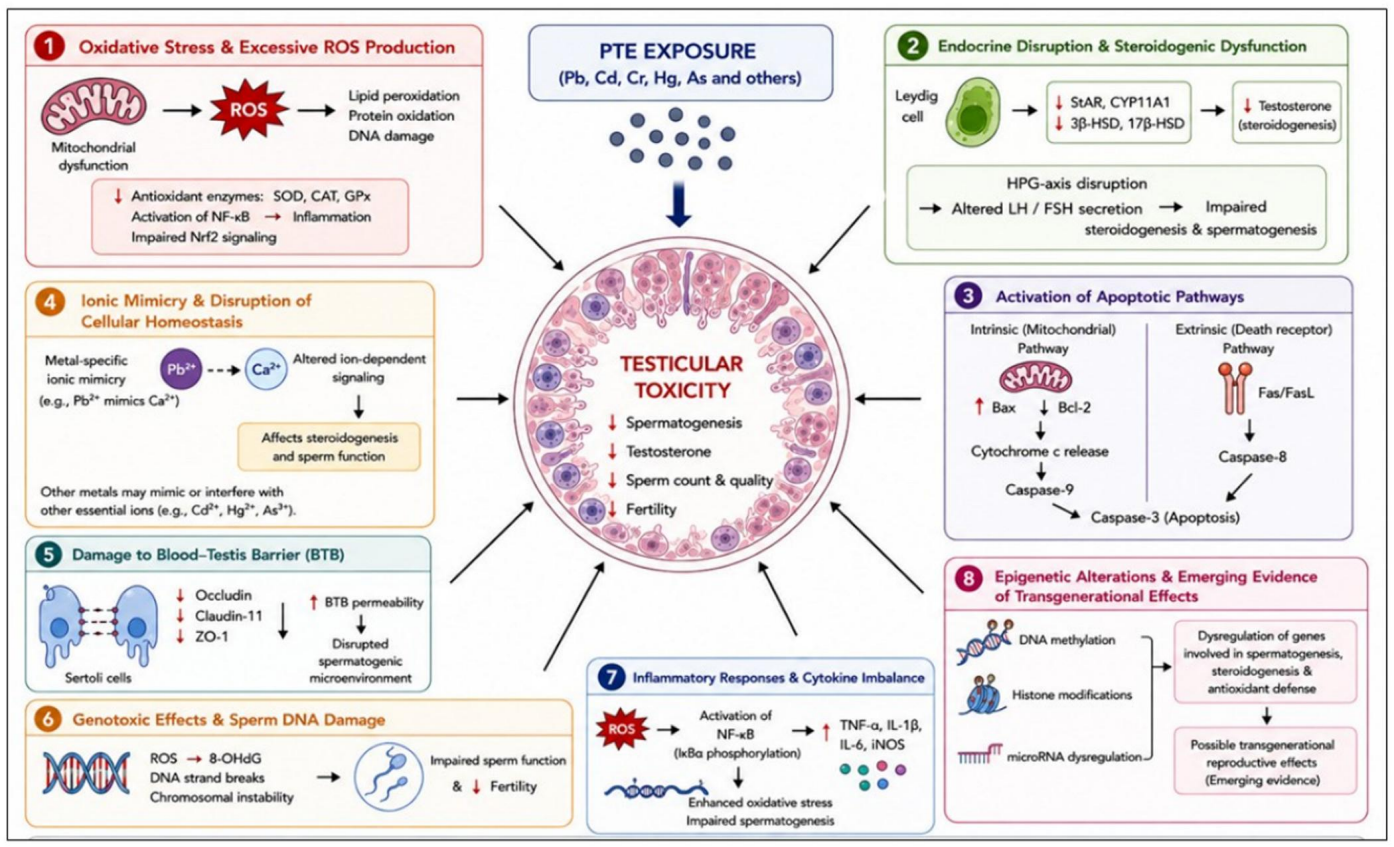
Schematic representation of the molecular and cellular mechanisms of PTE‐induced testicular toxicity leading to impaired spermatogenesis and male reproductive dysfunction.

### 2.3 Dose‐Dependent Effects of PTEs on Male Reproductive Toxicity

PTE‐induced male reproductive toxicity is strongly influenced by the dose, duration and pattern of exposure, which determine the severity and reversibility of toxic effects. High‐dose experimental studies have been invaluable in elucidating mechanisms such as oxidative stress, mitochondrial dysfunction, endocrine disruption, apoptosis and BTB damage [26]. However, these models may not accurately reflect the chronic, low‐level exposures encountered by humans through contaminated food, water, air and occupational settings. In contrast, prolonged exposure to environmentally relevant concentrations can induce cumulative oxidative stress, endocrine disturbances, epigenetic alterations and impaired spermatogenesis, even without overt histopathological changes [27]. Dose–response relationships are not always linear and very low concentrations may still produce measurable biological effects under specific conditions [28], higher doses generally produce more pronounced reproductive toxicity. Therefore, comparing low‐ and high‐dose effects is essential to improve the interpretation and translational relevance of experimental findings.

As shown in Table 1, increasing exposure dose generally amplifies the severity of reproductive injury, progressing from subtle molecular and endocrine alterations at low doses to pronounced structural and functional damage at higher doses. These findings highlight the importance of considering dose–response relationships when extrapolating experimental data to human reproductive risk assessment.

**Table 1:** Conceptual comparison of dose‐dependent patterns reported across experimental PTE studies.

Parameter	Low‐dose exposure (environmentally relevant)	High‐dose exposure (experimental/acute)	Representative references
Exposure pattern	Chronic exposure through contaminated food, water, air and occupational settings; gradual tissue accumulation	Acute or subacute exposure commonly employed in experimental toxicology studies	[27]
Oxidative stress	Mild but persistent ROS generation with initial activation of antioxidant defenses; prolonged exposure eventually exhausts antioxidant capacity	Excessive ROS production causing lipid peroxidation, mitochondrial dysfunction and antioxidant depletion	[29]
Endocrine effects	Mild reductions in testosterone with subtle alterations in LH and FSH	Marked suppression of steroidogenesis, testosterone depletion and HPG axis dysfunction	[30]
Blood–testis barrier (BTB)	Early disruption of Sertoli cell tight junctions and increased permeability	Severe BTB breakdown accompanied by extensive germ cell degeneration	[21]
Spermatogenesis	Progressive decline in sperm count, motility, morphology and fertility potential	Arrest of spermatogenesis with extensive germ cell loss and infertility	[31]
Histopathological alterations	Mild degeneration of seminiferous epithelium with subtle structural abnormalities	Seminiferous tubule degeneration, tubular atrophy, Leydig cell damage, necrosis and inflammatory infiltration	[24]
DNA damage and epigenetic alterations	Progressive oxidative DNA damage and epigenetic modifications	Extensive DNA fragmentation, chromosomal damage, apoptosis and potential transgenerational effects	[23]
Translational relevance	Closely resembles environmental and occupational human exposure	Valuable for mechanistic studies but may overestimate toxicity relative to typical human exposure	[25]

Abbreviations: BTB, blood–testis barrier; FSH, follicle‐stimulating hormone; HPG, hypothalamic–pituitary–gonadal; LH, luteinizing hormone; ROS, reactive oxygen species.

### 2.4 Major PTEs and Male Fertility

The following sections summarize the effects and underlying mechanisms associated with individual elements.

**Lead (Pb)**: Lead is a naturally existing metal used in lead‐acid batteries, pigments, coatings, metalworking operations, printing presses and is also alloyed as a shielding material [32]. Lead is commonly found as Pb^2+^ and can accumulate in both bone and soft tissues, including the testicular tissue. Acute lead poisoning occurs following exposure to high levels of lead. Chronic exposure can be lethal as lead gradually builds up in the body from continuous exposure to small amounts [33]. Animal studies consistently show that lead exposure reduces sperm quality and disrupts testicular architecture (Table 2).

**Table 2:** Lead‐induced changes in the testes.

Route/dose of lead	Experimental model/duration	Observed testicular effects	References
Oral Pb (CH_3_COO)_2_ (0.2%)	Wistar rats 70 days	Reduced sperm motility and count Increased abnormality in sperms Disorganized germinal epithelium of seminiferous tubules	[34]
Oral Pb (CH_3_COO)_2_ (100 mg/kg/day)	Kunming mice 3 weeks	Altered organization and degeneration of seminiferous tubules Partial to complete inhibition of spermatogenesis	[35]
Oral Pb (CH_3_COO)_2_ (50 g dissolved in 12 mL of 1 N HCl)	Albino Wistar rats 4 weeks	Testicular inflammation, edema and hydrocele formation	[36]
Oral Pb (CH_3_COO)_2_ (20 mg/kg/bw)	Albino rats 10 days	Increased DNA fragmentation and testicular histological changes Increased oxidative stress and lipid peroxidation Elevated lysosomal enzyme activity	[37]
Oral Pb (CH_3_COO)_2_ (0.15%)	Wistar rats 45 days	Reduced sperm count, testosterone concentration and antioxidant enzyme activity Significant decrease in testis weight	[38]

Abbreviations: bw, body weight; Pb (CH_3_COO)_2_, lead acetate.

### 2.5 Molecular and Cellular Mechanisms

Lead induces male reproductive toxicity through multiple interconnected molecular mechanisms (Figure 3). By mimicking Ca^2+^, Pb^2+^ can interfere with calcium‐dependent signaling and may enter cells through calcium‐permeable channels, disrupting calcium‐dependent signaling pathways essential for steroidogenesis and spermatogenesis. Pb accumulation in the testes and epididymis suppresses the expression of cytochrome P450 aromatase (CYP19) and estrogen receptor‐α (ERα), thereby impairing estrogen signaling and disrupting testosterone and estradiol homeostasis. Simultaneously, Pb‐induced oxidative stress activates NF‐κB and mitogen‐activated protein kinase (MAPK) signaling pathways, leading to increased production of pro‐inflammatory cytokines. Mitochondrial dysfunction further promotes apoptosis by increasing the Bax/Bcl‐2 ratio, facilitating cytochrome c release and subsequent caspase‐3 activation. These molecular perturbations culminate in degeneration of seminiferous tubules and Leydig cells, impaired spermatogenesis, decreased sperm quality and ultimately male infertility [39].

**Figure 3:**
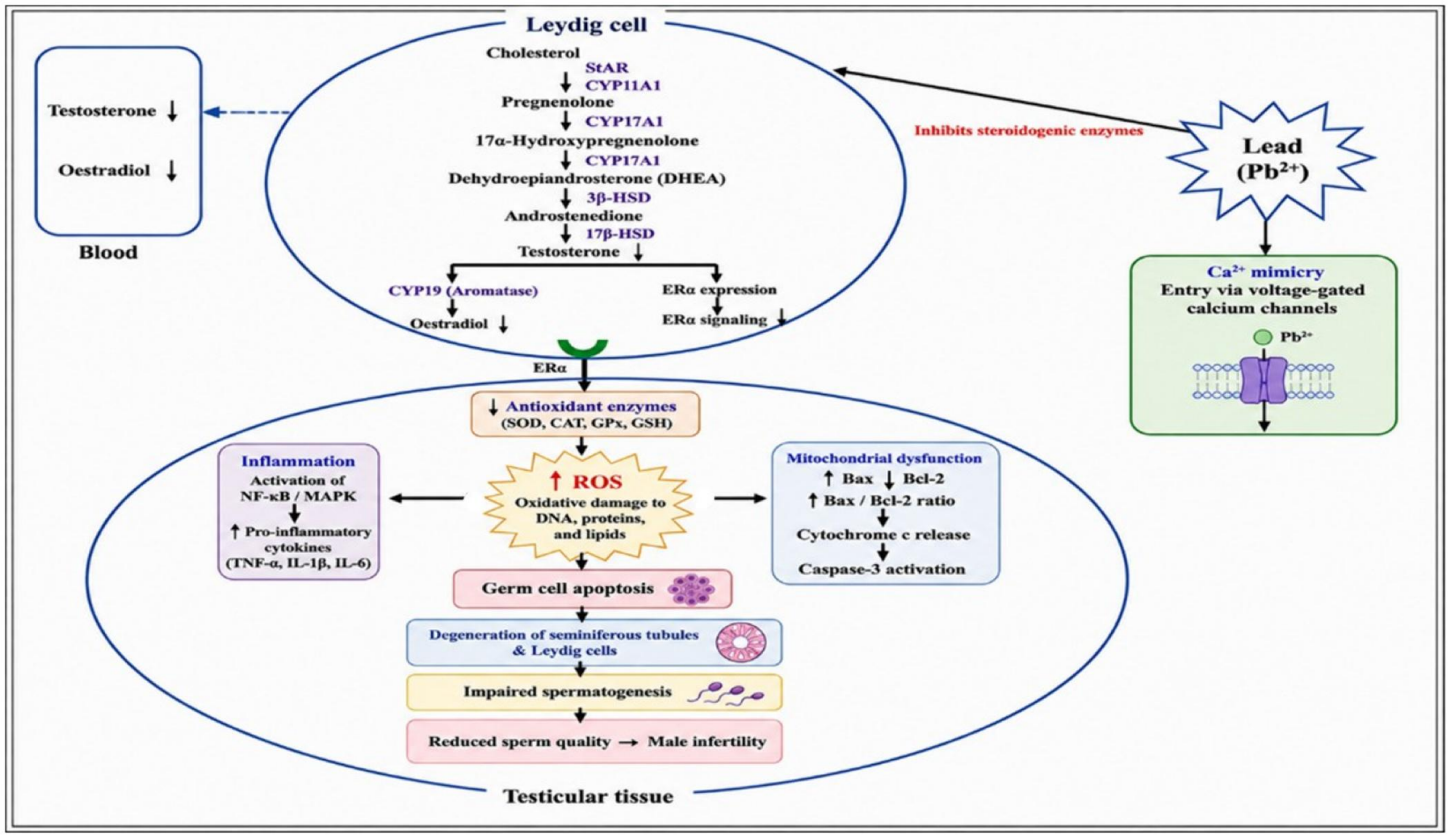
Proposed molecular pathways involved in Pb^2+^‐induced testicular toxicity. The schematic integrates the endocrine, oxidative, inflammatory and apoptotic mechanisms associated with Pb^2+^ exposure and their contribution to testicular dysfunction and male infertility.

**Cadmium (Cd)**: According to the Agency for Toxic Substances and Disease Registry (ATSDR), cadmium is considered the 7th most hazardous metal and is designated as a Group I carcinogen by the International Agency for Research on Cancer (IARC). It is often released into the environment through industrial emissions, battery manufacturing and phosphate fertilizers. Primary sources include cigarette smoke, contaminated food sources and polluted water [40]. Cadmium is regarded as one of the most potent testicular toxicants, as demonstrated by numerous rodent studies (Table 3).

**Table 3:** Cadmium‐induced changes in the testes.

Route/dose of cadmium	Experimental model/duration	Observed testicular effects	References
i.p. CdCl_2_ (1 mg/kg/bw/day)	Wistar Rats 1, 3, 10, and 21 days	Low SOD, CAT, GST activity High GSSG/GSH ratio	[41]
Single i.p. injection CdCl_2_ (2.5 mg/kg/bw)	Kunming mice 35 days	Reduced number of spermatogenic tubules Disorganized spermatogenic cells	[42]
Single i.p. injection CdCl_2_ (1.5 mg/kg/bw)	Swiss mice 7 days	Alterations in seminiferous tubular parameters and cell viability Vacuolization of seminiferous epithelium Seminiferous tubule degeneration	[43]
Oral CdCl_2_ (5 mg/kg/bw)	Wistar rats 28 days	Sperm count, motility and viability declined	[44]
Oral CdCl_2_ (5 mg/kg/bw)	Wistar rats 30 days	Reduced sperm count, motility and viability; abnormal morphology Atrophy of seminiferous tubules Disrupted testicular structure	[45]
Oral CdCl_2_ (2, 4, 8 mg/kg/bw)	Mice 8 weeks	Thinned germinal epithelium and abnormal tubules Suppressed spermatogenesis Altered testicular stroma	[46]
i.p. CdCl_2_ (1 mg/kg/bw)	Mice 7 days	Leydig cell ferroptosis Impaired autophagic flux Decreased testosterone synthesis Oxidative stress	[47]

Abbreviations: bw, body weight; CdCl_2_, cadmium chloride; CAT, catalase; GST, glutathione S‐transferase; GSSG/GSH, oxidized‐to‐reduced glutathione ratio; i.p., intraperitoneal; MDA, malondialdehyde; NO, nitric oxide; SOD, superoxide dismutase.

### 2.6 Molecular and Cellular Mechanisms

Cadmium can accumulate in testicular cells, including Sertoli cells, through metal transport pathways via Zrt‐ and Irt‐like protein 8 (ZIP8) and divalent metal transporter 1 (DMT1), making these cells a primary target of Cd‐induced testicular toxicity. Although Cd is not a redox‐active metal, it indirectly promotes oxidative stress by depleting reduced glutathione (GSH) and protein‐bound sulfhydryl groups, displacing Zn and Se from antioxidant enzymes, and suppressing the activities of SOD and CAT. These alterations enhance the generation of ROS, including superoxide anions and hydroxyl radicals, leading to lipid peroxidation, mitochondrial dysfunction and DNA damage [48].

In Sertoli cells, Cd disrupts BTB integrity by activating the transforming growth factor‐β3 (TGF‐β3)/MAPK signaling pathway, resulting in the downregulation of tight junction proteins, including claudin‐11, occludin, ZO‐1, and connexin‐43. The consequent disruption of the seminiferous microenvironment compromises germ‐cell development and spermatogenesis [48].

In Leydig cells, Cd impairs steroidogenesis by suppressing the expression of StAR, CYP11A1, 3β‐hydroxysteroid dehydrogenase type 1 (HSD3B1), cytochrome P450 17A1 (CYP17A1) and the luteinizing hormone/choriogonadotropin receptor (LHCGR), thereby reducing testosterone synthesis. Concurrently, Cd activates NF‐κB and c‐Jun N‐terminal kinase (JNK) signaling, together with mitochondrial apoptotic pathways, endoplasmic reticulum (ER) stress and autophagy, culminating in germ‐cell apoptosis, impaired spermatogenesis, reduced sperm quality and male infertility [49] (Figure 4).

**Figure 4:**
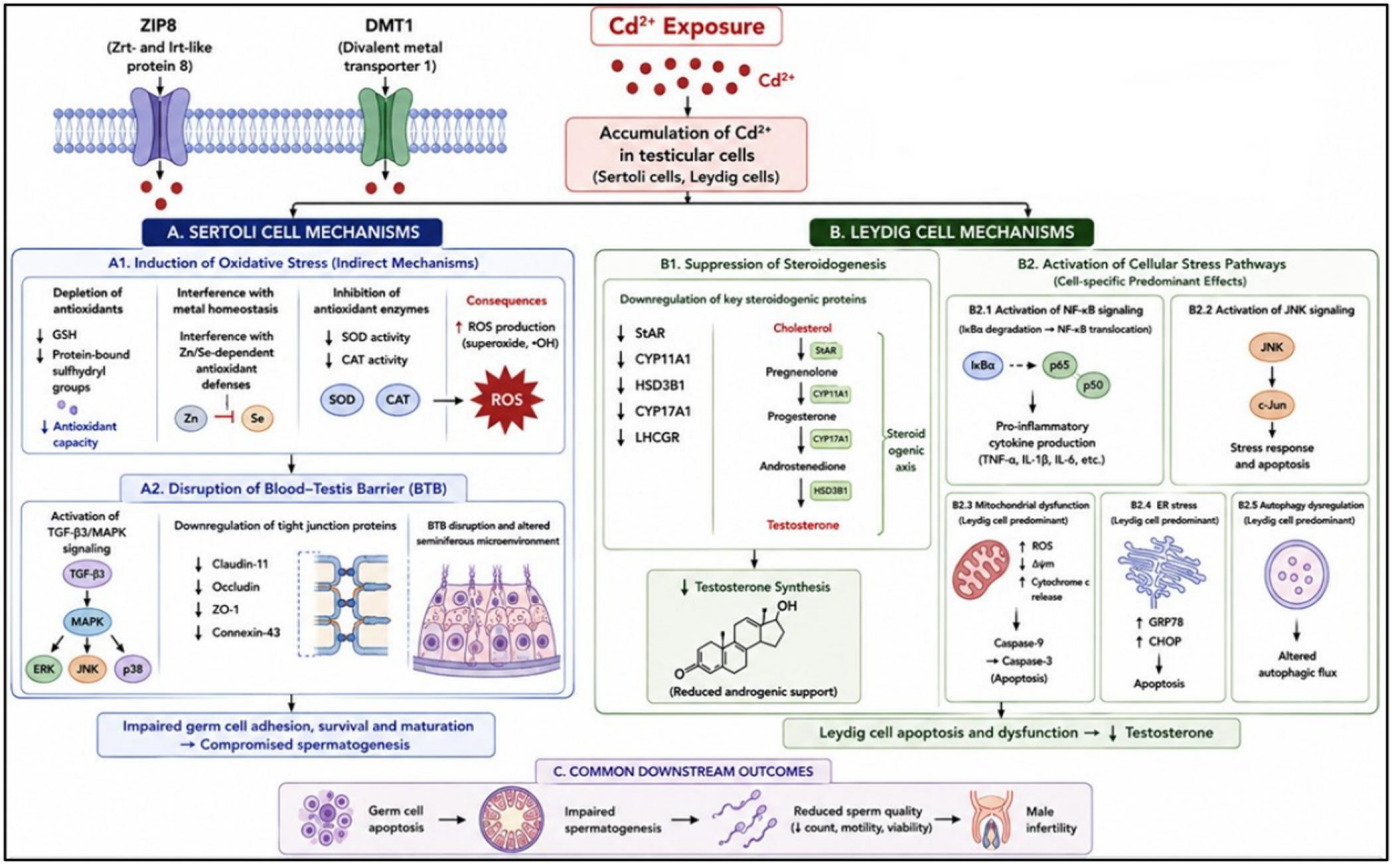
Schematic representation of the molecular mechanisms underlying cadmium‐induced male reproductive toxicity. Cd triggers oxidative stress, BTB disruption, endocrine dysfunction, inflammation and apoptosis, leading to impaired spermatogenesis and male infertility.

**Chromium (Cr)**: Chromium is a naturally occurring element present in the earth's crust and exists in several oxidation states ranging from Cr(II) to Cr(VI), of which hexavalent chromium (Cr(VI)) is the most toxic and biologically active form [50]. Owing to its high toxicity and widespread environmental contamination, the United States Environmental Protection Agency (EPA) has identified chromium as one of the most hazardous HMs.

Occupational exposure to Cr(VI) is common in industries such as electroplating, leather tanning, stainless steel manufacturing and pigment production, whereas the general population may be exposed through sources including cigarette smoke and automobile exhaust [51]. Cr(VI) exposure has been associated with a wide range of adverse health effects, including persistent skin and nasal irritation, contact dermatitis, emphysema, ulceration and carcinogenesis. At the cellular level, Cr(VI) enters cells through nonspecific anion channels, where it undergoes intracellular reduction reactions that generate ROS, initiating oxidative stress and subsequent cellular damage [52].

### 2.7 Molecular and Cellular Mechanisms

Chromium induces testicular toxicity primarily through oxidative stress and genotoxic mechanisms. Owing to its structural similarity to sulfate, Cr(VI) readily enters testicular cells via sulfate anion transporters and undergoes sequential intracellular reduction to Cr(V), Cr(IV), and Cr(III). This reduction process generates ROS and reactive chromium intermediates, leading to lipid peroxidation, protein oxidation, oxidative DNA lesions such as 8‐OHdG, DNA strand breaks and DNA–protein cross‐links. The resulting genomic damage activates the ataxia telangiectasia mutated (ATM)/ataxia telangiectasia and Rad3‐related (ATR)‐p53 signaling pathway, triggering mitochondrial apoptosis. In addition, ROS‐mediated oxidative stress impairs the expression and activity of key steroidogenic enzymes and damages Sertoli and Leydig cells, thereby reducing testosterone production and disrupting spermatogenesis [53]. Collectively, these molecular alterations culminate in increased germ‐cell apoptosis, impaired sperm quality and male infertility (Figure 5).

**Figure 5:**
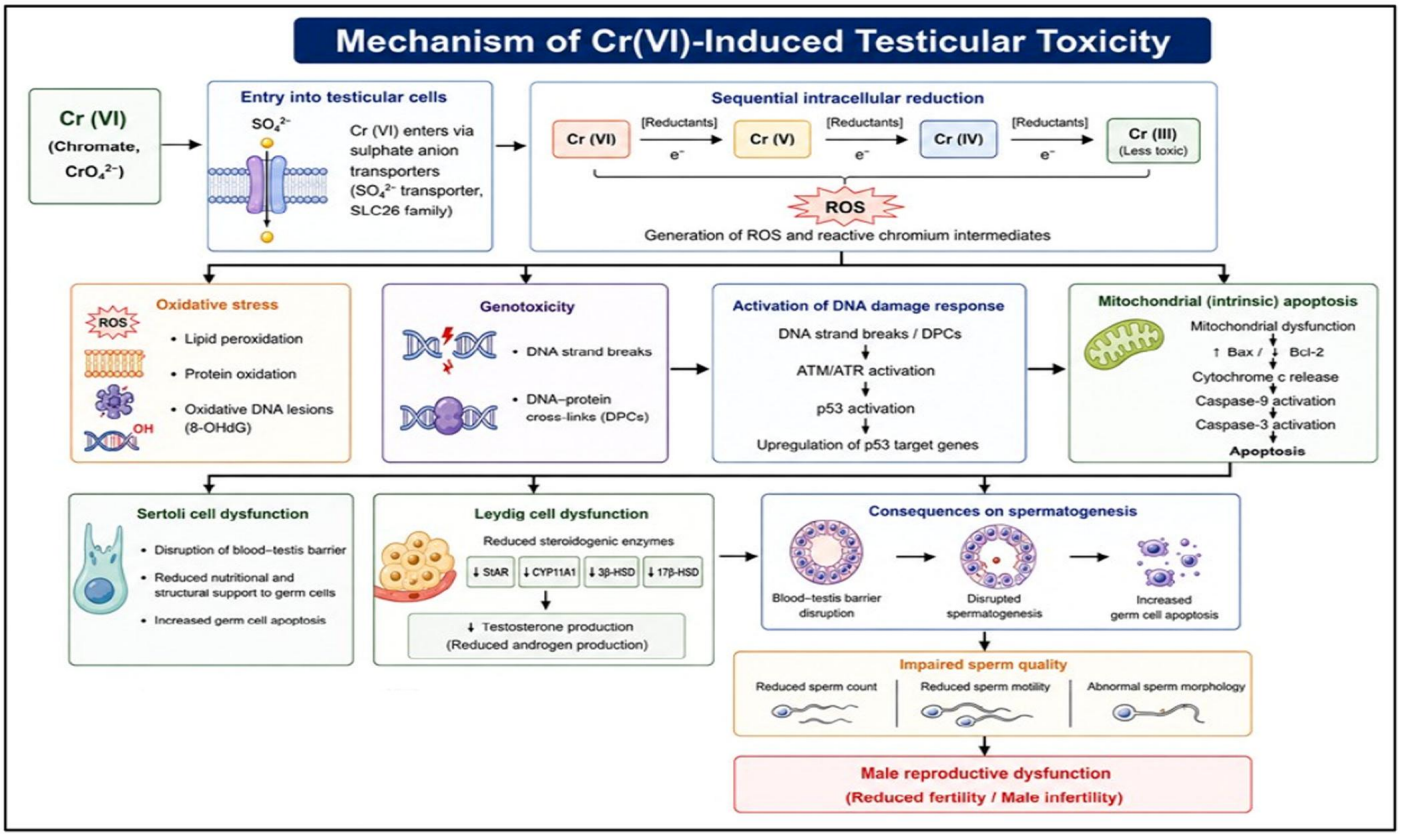
Schematic representation of the mechanism of Cr(VI)‐induced testicular toxicity, highlighting oxidative stress, genotoxicity, apoptosis, impaired spermatogenesis and male infertility.

Experimental investigations have identified chromium as an inducer of oxidative DNA damage and spermatogenic arrest (Table 4).

**Table 4:** Chromium‐induced changes in the testes.

Route/dose of chromium	Experimental model/duration	Observed testicular effects	References
Oral K_2_Cr_2_O_7_ (10.5 mg/kg/bw/day)	Rats 5 days/week for 10 weeks	Reduced SOD and GSH Degeneration of spermatogonia and Sertoli cells Atrophied disorganized seminiferous tubules	[54]
Oral K_2_Cr_2_O_7_ (10 mg/kg/bw)	Mice 30 and 45 days	Cr induced testicular histopathological alterations and impairment of testicular function	[55]
Oral K_2_Cr_2_O_7_ (8 mg/kg/bw)	Rats 42 days	Low testicular glucose level Decrease in the serum testosterone Testicular lesions and spermatogenic arrest Increase in cholesterol and triglyceride levels	[56]
i.p.K_2_Cr_2_O_7_ (1 and 2 mg/kg/bw)	Wistar rats15 days	Increased lipid peroxidation and metallothionein levels Increased DNA degradation Reduced CAT activity Germ cells apoptosis	[57]
Oral K_2_Cr_2_O_7_ (1.5 mg/kg/bw)	Mice 60 days	Low sperm count and motility Disrupted spermatogenesis	[58]
Oral CrO_3_ (10 mg/kg/bw)	Rats 90 days	High serum PSA concentration Damaged seminiferous tubules	[59]

Abbreviations: BTB, blood‐testis barrier; bw, body weight; CAT, catalase; CrO_3_, chromium trioxide; DNA, deoxyribonucleic acid; GSH, reduced glutathione; i.p., intraperitoneal; K_2_Cr_2_O_7_, potassium dichromate; PSA, prostate specific antigen; ROS, reactive oxygen species; SOD, superoxide dismutase.

**Mercury (Hg)**: Mercury is present in the Earth's crust in various chemical and physical forms. These include elemental (metallic) mercury, inorganic mercury and organic‐methyl mercury [2]. Human exposure to mercury primarily occurs through the consumption of seafood or sashimi particularly contaminated with methylmercury and to a lesser degree via dental amalgams, broken thermometers, fluorescent lamps, button batteries and skin‐lightening creams. Methylmercury and its compounds are the main sources of organic mercury in humans [60].

The most recognized mercury compound is likely HgCl_2_. This colorless compound is used in agriculture (as a fungicide), in industry (as a catalyst for vinyl chloride production) and as a precursor for other mercury‐related compounds. It is one of the main ingredients in skin brightening cosmetics which are used to treat freckles and skin spots. It causes a wide range of neurological, respiratory, immune, renal, reproductive and developmental effects [61].

### 2.8 Molecular and Cellular Mechanisms

Owing to its high affinity for sulfhydryl (–SH) groups, Hg readily binds to GSH and other thiol‐containing proteins, depleting intracellular antioxidant reserves and inactivating antioxidant enzymes [62]. These interactions impair mitochondrial respiration, reduce ATP production and promote excessive generation of ROS, thereby overwhelming the cellular antioxidant defense system. Consequently, the activities of key antioxidant enzymes, including SOD and CAT decrease, leading to enhanced lipid peroxidation, oxidative DNA damage and further mitochondrial dysfunction. The resulting mitochondrial injury triggers the intrinsic apoptotic pathway through cytochrome *c* release and subsequent caspase activation, culminating in germ‐cell apoptosis [63]. Simultaneously, Hg‐mediated activation of NF‐κB signaling exacerbates inflammatory responses, further contributing to testicular injury. In addition, Hg disrupts Leydig cell function by suppressing steroidogenesis and testosterone synthesis, thereby impairing the hormonal regulation of spermatogenesis. Collectively, these molecular and cellular alterations lead to degeneration of the seminiferous epithelium, impaired spermatogenesis, reduced sperm count, motility and viability and ultimately male infertility (Figure 6). Experimental evidence consistently identifies mitochondrial dysfunction and oxidative stress as the principal mechanisms underlying Hg‐induced reproductive toxicity [64] (Table 5).

**Figure 6:**
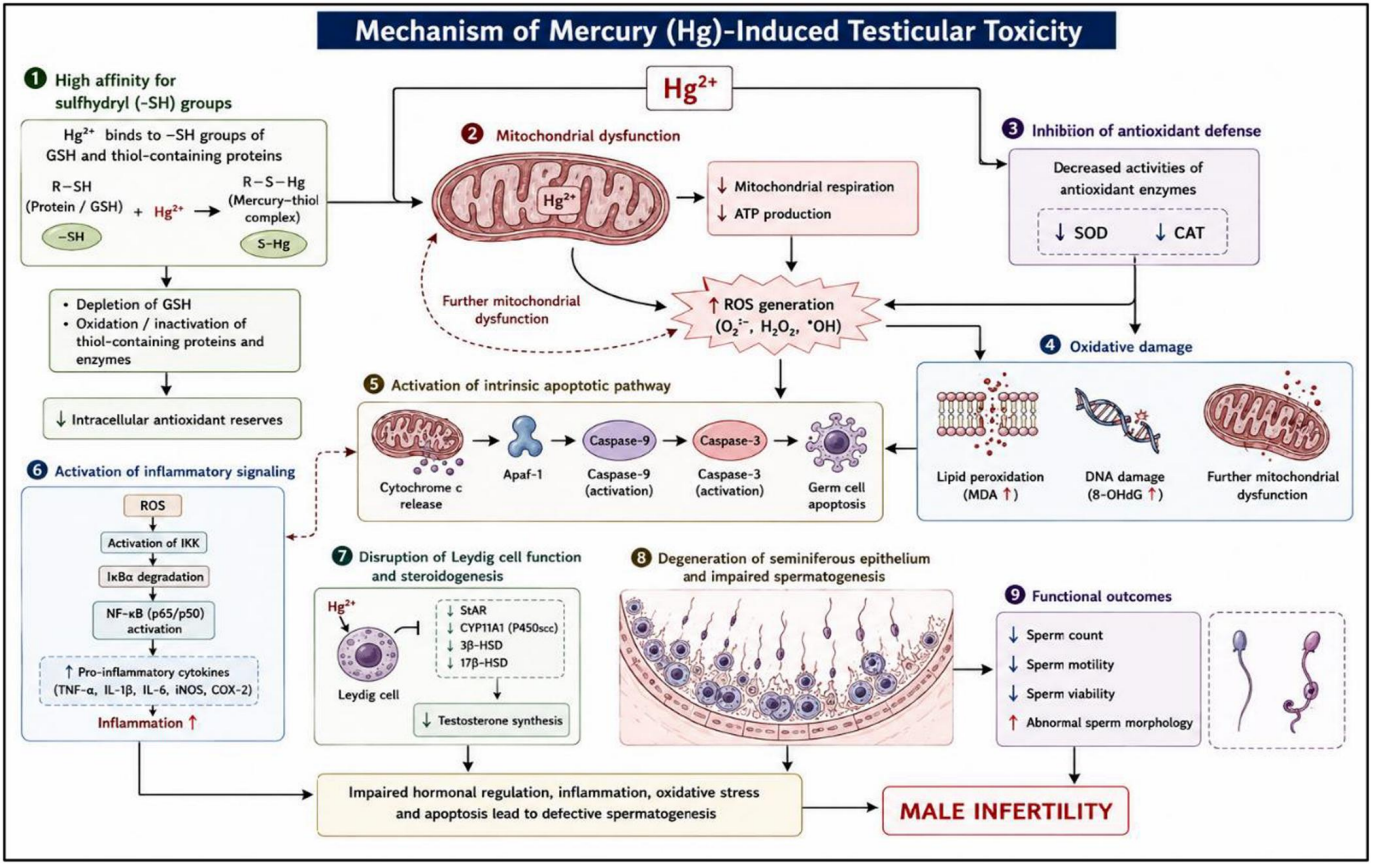
Proposed mechanisms of mercury induced testicular toxicity. Hg binding to thiol‐containing molecules triggers oxidative stress, mitochondrial dysfunction, ER stress, inflammation, endocrine disruption and Sertoli cell impairment, leading to germ‐cell apoptosis, impaired spermatogenesis and male infertility.

**Table 5:** Mercury‐induced changes in the testes.

Route/dose of mercury	Experimental model/duration	Observed testicular effects	References
Oral HgCl_2_ (40 mg/kg/bw)	Wistar rats 28 days	Degeneration and loss of germ cells Narrowing or blockage of seminiferous tubule lumen Enlargement and structural distortion of seminiferous tubules Basement membrane disruption with vacuole formation	[65]
Oral HgCl_2_ (0.4 mg/kg/bw)	Wistar rats 28 days	Significant reduction in spermatozoa concentration Loss of normal cellular arrangement and integrity of spermatogenic cells Formation of vacuolated areas within the seminiferous tubules	[66]
s.c. HgCl_2_ (5 mg/kg/bw)	Wistar rats 5 days	Fibrotic changes in seminiferous tubules Reduced spermatocyte and spermatozoa count	[67]
i.p. HgCl_2_ (1.23 mg/kg/bw)	Sprague Dawley rats 7 days	Interstitial edema and vascular dilation Tubular wall thinning with necrotic changes Increased inflammatory marker expression (TNF‐α, COX‐2)	[68]
i.p. HgCl_2_ (3 mg/kg/bw)	Kunming mice 7 days	Immunosuppression and fibrosis in testis High proportion of apoptotic cells, low serum testosterone	[69]
Oral HgCl_2_ (3 mg/kg/bw)	Albino rats 30 days	Reduction in sperm viability, motility, count and testosterone levels	[70]

Abbreviations: bw, body weight; COX‐2, cyclooxygenase‐2; HgCl_2_, mercuric chloride; i.p., intraperitoneal; s.c., subcutaneous; TNF‐α, tumor necrosis factor‐alpha.

**Arsenic (As)**: Arsenic is a poisonous metalloid element that is odorless, tasteless and present everywhere in nature. The most common forms of arsenic are inorganic arsenic, organic arsenic and arsine gas [71]. Inorganic arsenic is considered more harmful than its organic counterpart and can be found in two primary oxidation states: the trivalent form, arsenite (As^3+^), and the pentavalent form, arsenate (As^5+^) [72]. The International Agency for Research on Cancer (IARC) classifies arsenic as a Group‐I carcinogen, which also increases the risk of cancers in the bladder, lung, kidney and liver.

Its compounds are utilized in the pharmaceutical industry, wood preservation, agricultural chemicals such as pesticides, insecticides and herbicides, as well as in the mining, metallurgical, glass‐manufacturing and semiconductor sectors [73]. Furthermore, in the cosmetic industry, color pigments utilized in creating eye shadows often contain arsenic [74].

Humans can come into contact with arsenic naturally, through industrial sources or from accidental exposure. Drinking water can become contaminated from the use of arsenic‐based pesticides, natural mineral deposits or improper disposal of arsenic chemicals. Acute exposure to arsenic leads to nausea, vomiting, abdominal pain and severe diarrhea, whereas chronic exposure results in damage to multiple organs [75].

### 2.9 Molecular and Cellular Mechanisms

Following cellular uptake via aquaglyceroporins (AQPs), inorganic arsenic undergoes methylation by arsenic methyltransferase (AS3MT) to form monomethylarsonic acid (MMA) and dimethylarsinic acid (DMA), generating reactive intermediates that promote excessive ROS production. In addition, arsenite (AsIII) inhibits pyruvate dehydrogenase by interacting with lipoic acid‐dependent enzymes and disrupts the mitochondrial ETC, resulting in ATP depletion and further oxidative stress [76] (Figure 7).

**Figure 7:**
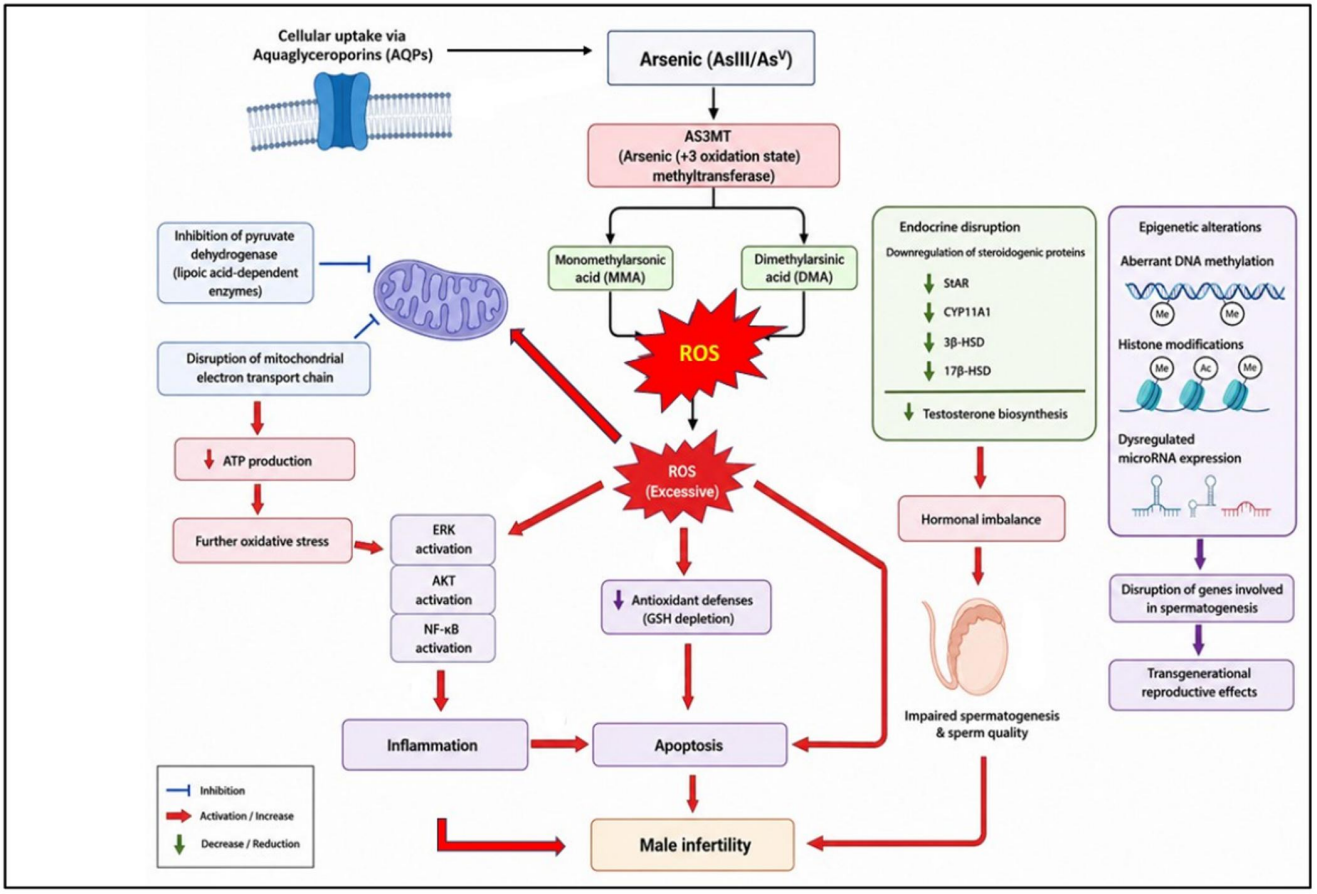
Molecular mechanisms of arsenic‐induced male reproductive toxicity.

The excessive generation of ROS depletes intracellular GSH, suppresses antioxidant defenses and activates Extracellular Signal‐Regulated Kinase (ERK), AKT, and NF‐κB signaling pathways [77], leading to inflammation and apoptosis. Arsenic also downregulates key steroidogenic proteins, including StAR, CYP11A1, 3β‐HSD, and 17β‐HSD, thereby reducing testosterone biosynthesis. Furthermore, arsenic induces epigenetic alterations, including aberrant DNA methylation, histone modifications and dysregulated microRNA expression, disrupting genes involved in spermatogenesis and potentially contributing to transgenerational reproductive effects. Collectively, these molecular events impair spermatogenesis, reduce sperm quality and ultimately lead to male infertility. Accordingly, chronic arsenic exposure has consistently been associated with impaired spermatogenesis and endocrine dysfunction in experimental models [78] (Table 6).

**Table 6:** Arsenic‐induced changes in the testes.

Route/dose of arsenic	Experimental model/duration	Observed testicular effects	References
Oral NaAsO_2_ 10 mg/L	Wistar rats PND 21–51	Lower sperm production, sperm count, motility and quality.	[79]
Drinking water NaAsO_2_ 10 mg/L	Wistar rats 56 days	Decreased daily sperm production and epididymal sperm count Enhanced sperm head abnormalities	[80]
i.p. (two consecutive injections) NaAsO_2_ 4 mg/kg/bw in 0.9% NaCl	Albino rats 28 days	Disorganized seminiferous tubules Low sperm production	[81]
Oral NaAsO_2_ 8 mg/kg/day	Rats 8 weeks	The sperm number, motility, viability and normal sperm morphology were significantly reduced	[82]

Abbreviations: bw, body weight; i.p., intraperitoneal; NaAsO_2_, sodium arsenite; PND, post natal day.

**Critical Appraisal of Experimental Evidence**: Although animal studies have provided valuable insights into PTE‐induced reproductive toxicity, the methodological quality of the available studies varies considerably owing to differences in experimental design, exposure protocols and outcome measures [83]. Most investigations employ rodent models, whose reproductive physiology differ from those of humans, limiting direct extrapolation of the findings.

Another important limitation is that most experimental studies investigate individual PTE despite environmental exposure commonly involving multiple PTEs. Such mixtures may produce additive, synergistic or antagonistic interactions that cannot be predicted from single‐PTE studies alone [84]. Consequently, current experimental models may underestimate the reproductive risks associated with real‐world exposure scenarios. Moreover, many studies use relatively high doses administered over short durations or by intraperitoneal injection, whereas human exposure is typically chronic, low‐dose and occurs through contaminated food, water or inhalation [85]. Future research should emphasize environmentally relevant chronic exposure models, standardized experimental protocols and longitudinal human studies to improve translational relevance.

### 2.10 Comparative Mechanisms of PTEs‐Induced Testicular Toxicity

Although PTEs discussed above impair male reproductive function, they differ in their primary molecular targets and the initiating molecular events differ substantially among individual metals. Despite these differences, the downstream consequences—including oxidative stress, mitochondrial dysfunction, inflammation, apoptosis, endocrine disruption, and impaired spermatogenesis—are remarkably similar, ultimately leading to male infertility.

Each metal also exhibits distinct cellular targets within the testes. Cadmium has a particularly pronounced effect on Sertoli‐cell function and BTB integrity, leading to germ cell apoptosis and impaired spermatogenesis [86]. In contrast, lead can substantially affect Leydig‐cell steroidogenesis and testosterone synthesis, thereby disturbing HPG axis function [30]. Cr(VI) has a strong genotoxic component involving oxidative DNA damage [53], whereas mercury can disrupt mitochondrial and thiol‐dependent cellular processes, impairing ATP production and reducing sperm motility [62]. Arsenic exerts broader toxicity by affecting multiple testicular cell types while inducing epigenetic alterations, including aberrant DNA methylation and histone modifications, which may contribute to long‐term reproductive dysfunction [78].

The mechanisms responsible for reproductive dysfunction also differ among metals. Lead toxicity is characterized by calcium mimicry, disrupting intracellular signaling and steroidogenesis. Cadmium has a particularly well‐characterized effect on BTB integrity through disruption of tight junction proteins [87]. Chromium (VI) is distinguished by its strong genotoxic potential resulting from intracellular reduction and oxidative DNA damage [88]. Mercury primarily inhibits protein function through irreversible binding to thiol‐containing enzymes, whereas arsenic is notable for inducing epigenetic dysregulation in addition to oxidative and inflammatory injury [63].

Overall, understanding the comparative mechanisms provides a comprehensive framework for interpreting PTE‐induced male reproductive toxicity and identifying potential therapeutic targets. Figure 8 summarizes the major molecular mechanisms underlying PTE‐induced male reproductive toxicity. Despite differences in their cellular entry and primary targets, Pb, Cd, Cr(VI), Hg, and As activate overlapping pathological pathways that culminate in impaired spermatogenesis and male infertility.

**Figure 8:**
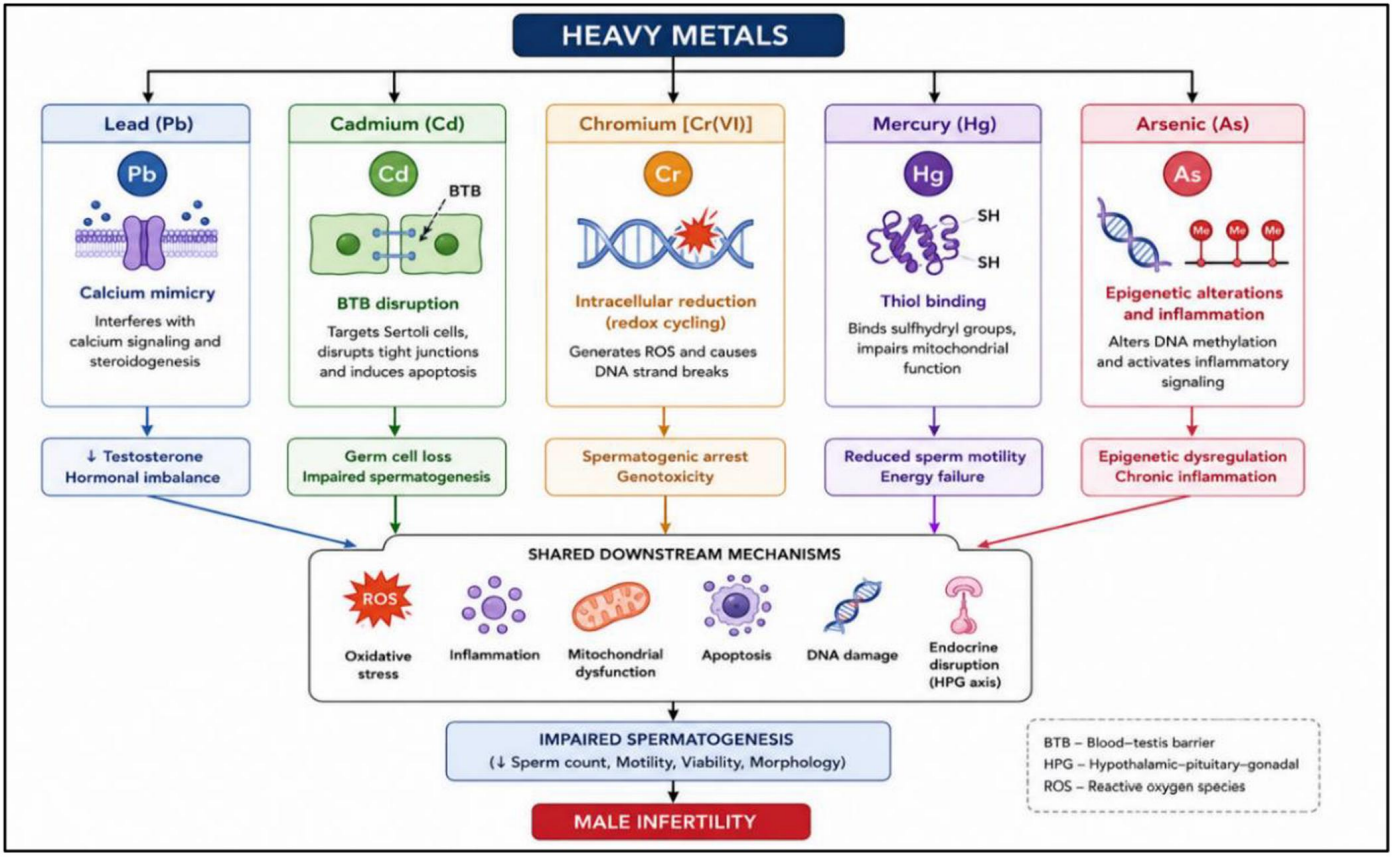
Comparative overview of the molecular mechanisms of PTE‐induced male reproductive toxicity.

### 2.11 Mixed‐Metal Exposure and Interaction Effects

In real‐world environmental and occupational settings, humans are rarely exposed to a single metal; instead, exposure typically occurs as complex mixtures through contaminated air, water, food and industrial activities. Consequently, mixed‐metal toxicity provides a more realistic representation of human exposure than studies evaluating individual metal [89, 90]. Depending on the metal combination, dose and duration of exposure, interactions may be additive, synergistic or antagonistic. Experimental evidence indicates that metal mixtures frequently exacerbate oxidative stress by increasing ROS generation, lipid peroxidation, mitochondrial dysfunction, DNA damage, inflammation and depletion of antioxidant defenses [91].

Although synergistic and additive interactions are more commonly reported, antagonistic interactions may also occur through competition for cellular transporters, binding proteins or metabolic pathways, thereby reducing the bioavailability or toxicity of specific metals [92]; for example, essential elements such as Zn and Se can mitigate Pb‐ and Cd‐induced toxicity by competing for binding sites and enhancing antioxidant defenses [93]. Nevertheless, most experimental studies continue to evaluate individual metal, underscoring the need for reproductive toxicology studies using environmentally relevant metal mixtures to better reflect real‐life exposure scenarios and improve human health risk assessment [94].

### 2.12 Implications of PTE Exposure for Male Fertility: Evidence From Human Epidemiological Studies

Human epidemiological studies provide substantial evidence linking PTE exposure with impaired male reproductive health. The key findings from representative epidemiological studies investigating the effects of PTEs on male fertility are summarized in Table 7.

**Table 7:** Human epidemiological studies examining PTE exposure and male fertility outcomes.

Study design	Study population	Exposure assessed	Methods	Major findings	Conclusion	References
OCCUPATIONAL EXPOSURE STUDIES						
Cross‐sectional comparative study	71 welders versus 71 controls	Welding fumes (Pb, Al, Mn, Zn)	Metal analysis, hormone assays, SOD measurement, IIEF‐5 questionnaire	↑ Pb, Al, Mn levels ↑ FSH/LH ↓ Testosterone, SOD, libido and erectile function in welders	Metal fume exposure impairs male reproductive health	[95]
Systematic review and meta‐analysis	Men from human studies	Cd (blood, semen, urine)	Pooled analysis of published studies	↓ Sperm count and motility ↓ Testosterone Altered sex hormones	Cd exposure impairs male reproductive health	[96]
Cross‐sectional comparative study	40 copper smelter workers and 40 controls	Cu, Pb, As	Biomonitoring and hormonal assays	↑ Cu, Pb, As, FSH and LH ↓ Testosterone ↑ Oxidative stress (MDA)	Combined metal exposure impairs male reproductive hormones through oxidative stress	[97]
Case control study	60 exposed men versus 30 controls	Cd, Pb, Cr	Semen and metal analysis	↑ ROS, MDA and sperm DNA fragmentation ↓ Antioxidant enzymes and acrosome integrity ↑ Cd, Pb and Cr levels	Occupational exposure impairs sperm function through oxidative stress	[98]
GENERAL POPULATION STUDIES						
Scoping review (PRISMA 2020)	Review of 21 studies on heavy metal–exposed men	Pb, Cd, Hg, As	Literature review (PECOS criteria used)	Pb and Cd: ↓semen quality As: Oxidative stress ↑ and apoptosis Hg: Variable reproductive effects	Heavy metal exposure impairs semen quality and contributes to male infertility	[27]
Cross‐sectionalstudy	1247 men	Cd	ICP‐MS	As, Cd, Cu, Se and Sn: May impair male fertility Zn: May improve sperm concentration	Inverse correlation between Cd levels and total sperm motility	[99]
BIOMONITORING STUDIES						
Cross‐sectional comparative study	60 smokers and 20 non‐smokers	Pb and As in serum and seminal plasma	Semen analysis (WHO 2010), AAS	Smokers had poorer semen quality than nonsmokers ↑ Pb and As levels were associated with impaired semen parameters	Smoking‐associated Pb and As exposure may contribute to impaired male reproductive function	[100]
Cross‐sectional study	394 Chinese men	Urinary metals (13 trace/heavy metals)	Urine metal analysis, semen analysis, regression	↑ Ni: poorer sperm morphology ↑ Se: better sperm count	Ni may harm, while Se may improve semen quality	[101]
Case control study	Men with poor semen quality (*n* = 61) and controls (*n* = 55)	Cd and Pb in seminal plasma	ICP analysis of seminal plasma	↑ Cd and Pb levels in poor‐quality semen	Elevated seminal Cd and Pb levels were associated with poor semen quality	[102]

Abbreviations: AAS, atomic absorption spectrophotometry; Al, aluminum; As, arsenic; Cd, cadmium; Cr, chromium; Cu, copper; FSH, follicle‐stimulating hormone; Hg, mercury; ICP‐MS, inductively coupled plasma mass spectrometry; IIEF‐5, International Index of Erectile Function‐5; LH, luteinizing hormone; MDA, malondialdehyde; Mn, manganese; Ni, nickel; Pb, lead; ROS, reactive oxygen species; Se, selenium; Sn, tin; SOD, superoxide dismutase; Zn, zinc; ↑, increased; ↓, decreased.

Although most epidemiological studies report associations between PTE exposure and impaired semen quality, several limitations should be considered. Many studies are cross‐sectional in design and therefore cannot establish causality. In addition, sample sizes vary considerably and differences in exposure assessment methods, study populations and adjustment for confounding factors such as smoking, diet and occupational exposures may contribute to inconsistent findings.

Despite these limitations, epidemiological evidence consistently supports an association between PTE exposure and impaired male reproductive health, including reduced sperm quality and fertility potential. Moreover, PTE exposure may have implications beyond male fertility, potentially affecting embryo development, pregnancy outcomes and offspring health through sperm DNA damage and epigenetic alterations. These findings highlight the importance of minimizing environmental and occupational exposure to PTEs.

The major reproductive and public health implications of PTE exposure are outlined below:

1. **Decline in Sperm Quality**: Epidemiological studies consistently associate PTE exposure with impaired semen quality, altered reproductive hormone profiles and reduced fertility. They also report associations between occupational or environmental metal exposure and increased rates of oligospermia and infertility [103].
2. **Hormonal Imbalance**: PTEs interfere with Leydig cell function and the HPG axis. Reduced testosterone and altered LH/FSH secretion impair spermatogenesis and may contribute to sexual dysfunction and decreased libido [104].
3. **Testicular and Accessory Gland Damage**: Structural alterations in seminiferous tubules, Sertoli cells and the epididymis impair sperm maturation and storage. Chronic exposure may lead to testicular atrophy, reduced seminal fluid quality and subfertility.
4. **Transgenerational Effects**: PTE‐induced epigenetic alterations, including DNA methylation, histone modifications and microRNA dysregulation, may be transmitted to offspring causing persistent reproductive effects, although evidence for true transgenerational inheritance remains limited and is derived predominantly from animal studies [105].
5. **Public Health and Socio‐economic Implications**: PTE‐induced male infertility contributes to the growing global burden of subfertility and infertility. Populations exposed through industrial pollution or contaminated food and water sources face increased reproductive risks [106]. Environmental monitoring, early detection and mitigation strategies are essential to reduce long‐term societal impacts.

### 2.13 Molecular Mechanisms Underlying Protective Strategies Against PTE‐Induced Male Reproductive Toxicity

PTE‐induced male reproductive toxicity results from interconnected mechanisms which impair spermatogenesis and male fertility, highlighting the need for interventions that target multiple pathways. Several strategies exist for their removal from both the environment and the human body. Some of them are discussed below.

### 2.14 Antioxidant Supplementation

Oxidative stress is the principal mediator of PTE‐induced testicular injury, making antioxidants one of the most widely investigated therapeutic strategies. Vitamins C and E, melatonin, N‐acetylcysteine and coenzyme Q10 directly scavenge ROS, replenish intracellular GSH and restore the activities of antioxidant enzymes, including SOD, CAT and GPx. Consequently, lipid peroxidation, oxidative DNA damage and mitochondrial dysfunction are attenuated, preserving Sertoli, Leydig and germ cell function [107]. Vitamin C also regenerates oxidized vitamin E, enhancing membrane stability and sperm motility. However, poor bioavailability, dose‐dependent variability and limited clinical evidence restrict the therapeutic translation of antioxidant supplementation [108].

### 2.15 Micronutrient Support and Cellular Signaling

Essential micronutrients such as zinc and selenium strengthen endogenous antioxidant defenses and support normal testicular function. Zinc competes with toxic metals for cellular uptake, whereas selenium acts as a cofactor for GPx, collectively reducing oxidative damage [93]. These micronutrients also preserve steroidogenic proteins, including StAR, CYP11A1 and 3β‐HSD, thereby maintaining testosterone synthesis and spermatogenesis. Furthermore, activation of the Nrf2 pathway and suppression of NF‐κB signaling enhance antioxidant gene expression while reducing inflammatory cytokine production. Nevertheless, excessive supplementation may disturb trace element homeostasis and should therefore be carefully optimized.

### 2.16 Phytochemicals and Flavonoids

Phytochemicals, particularly flavonoids and other polyphenolic compounds, have emerged as promising candidates for mitigating PTE‐induced testicular injury because of their antioxidant, anti‐inflammatory and cytoprotective properties. Experimental studies have investigated compounds such as quercetin, curcumin, resveratrol and epigallocatechin gallate (EGCG), which may counteract oxidative stress, inflammatory signaling, mitochondrial dysfunction and apoptosis associated with PTE exposure [109, 110, 111]. These findings suggest that flavonoid‐rich and other phytochemical‐based interventions may provide complementary protection against PTE‐induced reproductive toxicity, although their efficacy, bioavailability, optimal dosage and translational relevance require further investigation.

### 2.17 Emerging Nutraceutical‐Based Interventions

Although several phytochemicals discussed above have nutraceutical potential, inositols represent an emerging complementary approach to PTE‐induced reproductive toxicity. In male mice, myo‐inositol (MI) attenuated Cd‐induced oxidative and inflammatory damage, restored GSH and testosterone levels and improved testicular architecture and claudin‐11 expression. Co‐administration with seleno‐l‐methionine further improved several Cd‐induced alterations, supporting the potential of complementary nutraceutical combinations, although formal synergistic effects require specific interaction studies [112].

Given the multifactorial nature of PTE toxicity, combining nutraceuticals with antioxidant or anti‐inflammatory interventions may provide broader protection by targeting oxidative stress, inflammation and endocrine disturbances. However, dose, bioavailability, safety and therapeutic interactions require further investigation before clinical translation. Such approaches should complement, rather than replace, exposure reduction through appropriate environmental and agricultural practices and healthy dietary habits [113].

The interaction between nutraceuticals and the gut microbiota represents another emerging area. Nutraceuticals can modify microbial composition and function, while microbial metabolism may influence their bioavailability and activity [114]. Because PTE exposure may also disturb intestinal homeostasis and systemic inflammation, microbiota modulation could potentially influence downstream reproductive toxicity. However, direct evidence remains limited, warranting studies integrating nutraceuticals, gut microbiota, PTE burden and reproductive outcomes.

Future preventive or therapeutic strategies should therefore consider interactions among nutraceuticals, gut microbial metabolism and conventional interventions that may influence the bioavailability and efficacy of protective compounds.

### 2.18 Chelation Therapy and Lifestyle Interventions

Chelating agents such as ethylenediaminetetraacetic acid (EDTA), meso‐2,3‐dimercaptosuccinic acid (DMSA) and 2,3‐dimercapto‐1‐propanesulfonic acid (DMPS) reduce the body burden of chelatable PTEs by forming stable metal‐chelate complexes that facilitate renal excretion, thereby reducing systemic exposure to chelatable metals [115]. As chelation does not reverse established oxidative or mitochondrial damage and may also deplete essential trace elements, it is often more effective when combined with antioxidants or phytochemicals. Lifestyle interventions, including minimizing occupational exposure, consuming uncontaminated food and water, smoking cessation and antioxidant‐rich diets, further reduce cumulative PTE exposure and complement pharmacological therapies [116]. However, their effectiveness largely depends on sustained adherence and early implementation.

Overall, protective strategies mitigate PTE‐induced male reproductive toxicity through complementary mechanisms involving restoration of redox homeostasis, modulation of Nrf2‐ and NF‐κB‐dependent signaling, preservation of mitochondrial function and steroidogenesis, maintenance of BTB integrity and reduction of tissue metal burden. Future studies should focus on standardized experimental models, clinically relevant exposure scenarios and well‐designed human studies to facilitate translation of these promising strategies into effective interventions. Importantly, preventive measures aimed at reducing environmental and occupational exposure to PTEs should remain the primary approach for preserving male reproductive health.

## 3 Conclusion

PTE contamination remains an important environmental and public health concern with significant implications for male reproductive function. Although considerable progress has been made in understanding their effects, important knowledge gaps remain regarding chronic low‐dose exposure, mixed‐metal toxicity and long‐term reproductive outcomes in humans.

Future research should emphasize environmentally relevant exposure models, standardized experimental approaches and well‐designed epidemiological studies to improve translational relevance. In addition, the identification of sensitive biomarkers and the development of effective preventive and therapeutic interventions will be essential for reducing the reproductive burden associated with PTE exposure.

## Author Contributions

Sakshi Yadav wrote and edited the manuscript. P. J. John proofread and evaluated the manuscript. Both authors read and approved the final manuscript.

## Conflicts of Interest

The authors declare no conflicts of interest.

## Generative AI Statement

The authors declare that no generative artificial intelligence (AI) tools were used in the conception, preparation, writing, editing or creation of the manuscript. The manuscript was entirely prepared and reviewed by the authors, who take full responsibility for its content, accuracy and integrity.

## Data Availability

No new data were created or analyzed in this study. Data sharing is not applicable to this article.
